# Multidisciplinary care for patients with epidermolysis bullosa from birth to adolescence: experience of one Italian reference center

**DOI:** 10.1186/s13052-022-01252-3

**Published:** 2022-04-12

**Authors:** Chiara Retrosi, Andrea Diociaiuti, Cristiana De Ranieri, Marialuisa Corbeddu, Claudia Carnevale, Simona Giancristoforo, Maria Rosaria Marchili, Guglielmo Salvatori, Marta Luisa Ciofi degli Atti, May El Hachem, Massimiliano Raponi

**Affiliations:** 1 Dermatology Unit and Genodermatosis Unit, Genetics and Rare Diseases Research Division, Bambino Gesù Children’s Hospital, IRCCS, Rome, Italy; 2 Clinical Psychology Unit, Bambino Gesù Children’s Hospital, IRCCS, Rome, Italy; 3 Pediatric Emergency Department, Bambino Gesu’ Children’s Hospital, IRCCS, Rome, Italy; 4 Neonatal Intensive Care Unit and Human Milk Bank, Department of Neonatology, Bambino Gesù Children’s Hospital, IRCSS, Rome, Italy; 5 Clinical Epidemiology Unit, Bambino Gesù Children’s Hospital, IRCCS, Rome, Italy; 6 Health Directorate, Bambino Gesù Children’s Hospital, IRCCS, Rome, Italy

**Keywords:** Inherited epidermolysis bullosa, Therapeutic education plan, Genodermatosis, Squamous cell carcinoma, Chronic wounds

## Abstract

**Background:**

Epidermolysis bullosa (EB) is a disabling and chronic genodermatosis characterized by mucocutaneous fragility with blister formation after minimal trauma. Severity ranges between very mild forms to extremely severe or lethal subtypes. Depending on disease subtypes, blisters may be localized also in larynx, bladder, esophagus, and most frequent disease complications are malnutrition, chronic anemia, osteoporosis, limb contracture and early development of squamous cell carcinomas. EB is classified into four major groups: EB simplex (EBS), junctional EB (JEB), dystrophic EB (DEB) and Kindler EB (KEB). No specific treatment is available; however, a multidisciplinary management is mandatory in order to treat the lesions, to prevent complication, and to give a psychological support to the patient and family members.

**Objective:**

To report the experience on a therapeutic education plan of an Italian reference center for epidermolysis bullosa in the last 30 years.

**Methods:**

In our study we included all patients with EB from 1990 to the present, dividing them into three age groups (< 5 years, > 5–12 years and > 12–18 years). The therapeutic plan involved all multidisciplinary team members, since born until adolescence. The multidisciplinary team has been progressively established; the dermatologists act as patient case manager, in collaboration with the pediatrician, endocrinologist, dietician, dentist, plastic surgeon, digestive surgeon, geneticist, psychologist and a dedicated nurse. Other dedicated specialists are involved upon patient needs.

**Results:**

Two hundred fifteen patients have been recruited and followed in our hospital since 1990. One hundred forty patients (65%) are on follow-up, 27 patients (13%) died and only 11 (5%) were lost to follow-up. Our patients manifested the specific complications related to their EB subtype in keeping with the data reported in the literature. Eighteen (8%) patients affected with JEB severe died within the first year of life, 9 patients (5%) died for squamous cell carcinoma in adulthood and were affected with recessive DEB; only 1 patient died for squamous cell carcinoma at the age of 16.

**Conclusions:**

An adequate management of EB patients require a multidisciplinary approach with an educational plan to guarantee an appropriate treatment and to support and accompany patients and their families since birth along life. The dynamic educational plan adopted in our hospital showed good clinical and psychological outcome in our population, allowing adherence to treatment, reducing the frequency of complications and improving life expectancy and quality of life.

## Background

**Fig. 1:**
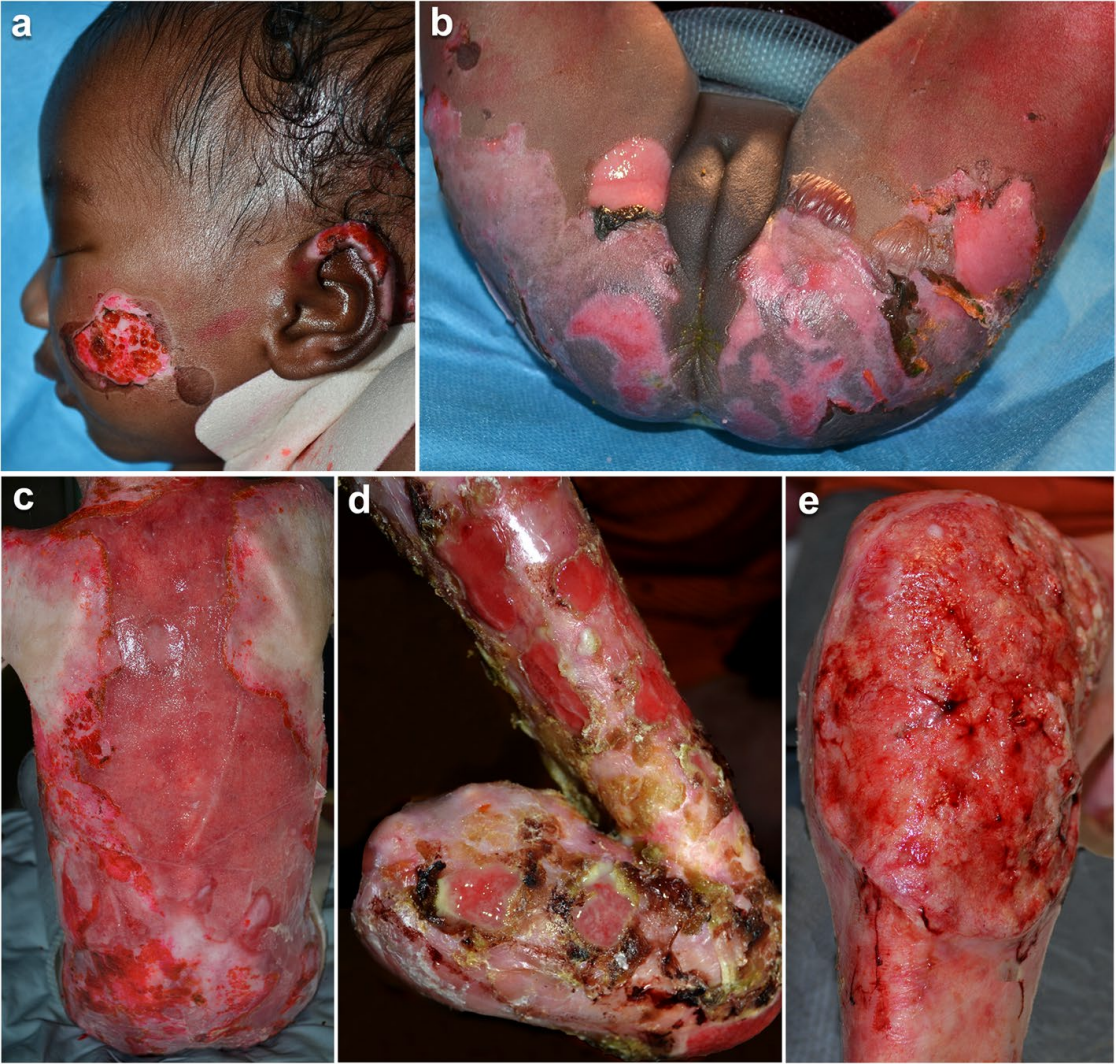
Epidermolysis bullosa clinical features. Erosions on the left cheek and ear in a female newborn affected with junctional epidermolysis bullosa (JEB) (**a**), similar lesions are extended on the buttocks, and genital area in the same patient (**b**); Wide and erosion on the back of a girl aged 8 years and affected with recessive dystrophic epidermolysis bullosa (**c**); Mitten deformity due to complete fusion of the fingers, and chronic erosions characterized by numerous crusts and exuberant tissue on the hand and forearm of a 17-years-old boy with recessive dystrophic epidermolysis bullosa (**d**); a large squamous cell carcinoma on the left knee of the same patient (**e**)

Epidermolysis bullosa (EB) is a clinically and genetically heterogeneous group of diseases characterized by mucocutaneous fragility with blister formation after minimal trauma. They are classified into four major types: EB simplex (EBS), junctional EB (JEB), dystrophic EB (DEB) and Kindler EB (KEB), and more than 30 subtypes [1]. EB subtypes are genetically and clinically heterogeneous and include a wide range of severity. They manifest generally at birth with blisters, erosions on the skin and oral mucosa. They are painful and the course is chronic. Extracutaneous involvement and complications (infections, scarring, pseudosyndactyly, joint contractures, corneal erosions and opacity, microstomia, dental caries, ankyloglossia, severe anemia, osteoporosis, early onset of muco-cutaneous squamous cell carcinomas etc.) are present in several EB forms, leading to early death in some EB subtypes (Fig. 1).

The diagnosis is suspected on clinical features and should be confirmed by a skin biopsy for immunofluorescence antigen mapping (IF), electron microscopy (in selected cases), and genetic-molecular testing. The relevance and specificity of each technique depends on the specific subtypes of epidermolysis bullosa: electron microscopy has higher sensitivity in EBS diagnosis, while IFM is best method in predicting severe JEB and RDEB cases, genetic testing confirms all diagnosis including some special EB subtypes, in addition, it distinguishes dominant from recessive DEB. The role of the genetician is important to administer genetic counselling and to inform the parents about the possibility to perform prenatal diagnosis.

EB highly affects the quality of life because: (i) it manifests frequently since birth, (ii) it is chronic, (iii) it does not have a curative treatment, (iv) it is painful and disabling, (v) it requires daily management, time consuming, (vi) it is often multisystemic, (vii) it is frequently characterized by reduced life expectancy, and (viii) results in severe esthetical damage (Fig. 1). In addition, the treatment is symptomatic and based on daily skin care and specific treatments for complications [2–4]. Complete medication of skin wounds may takes hours and be extremely painful requiring analgesia. The management of skin lesions varies upon the clinical manifestation. Before discharging the patient, parents/caregivers should be given a specific and personalized training in order to guarantee an appropriate treatment at home. Quality of life has been assessed by generic and specific questionnaires for EB, and reported in the literature [5–8]. In this manuscript, we describe the experience of our reference center for EB in the last 20 years, focusing on therapeutic educational plan specifically targeting three different age groups.

## Methods

### Patient population

We included in the study all patients with EB diagnosed in our reference center from 1990 onwards. Patients undergo at least annual follow-up with a multidisciplinary medical and/or surgical approach, diagnostic investigations and specific treatment.

### Therapeutic plan

The patients have been classified in 3 age groups: < 5 years, > 5–12 years and > 12–18 years, due to the wide variability of EB age-related features, encountered problems, difficulties on disease management and daily life, as well as self-perception, and interactions with family members. Dedicated dermatologist and nurse with the support of a psychologist in most cases deliver the therapeutic education. We inform the parents/caregivers to create a relaxing context, to prepare all required materials for dressing, and to administer analgesic drugs to the patient prior to medication.

The dermatologist and the nurse teach the parents how to handle the baby and to breastfeed him/her, how to remove the dressings avoiding trauma, to break the blister roof, and to remove the crusts in order to reduce itching, and to recognize wound characteristics, the respective management and dressing, to dress the fingers in patients with recessive dystrophic EB to delay syndactyly.

An appropriate wound management is mandatory. Specific advanced dressings should be used to i) improve healing, ii) prevent further lesions, iii) treat existing wounds and prevent/treat infection, iv) limit/delay complications such as contractures, squamous cell carcinoma, v) improve adherence to treatment. It is recommended to lance new blisters with a finger prick lancet or a sterile needle and drained to prevent their expansion, leaving the blister roof in place to reduce infection risk and pain. Wound management strategies should consider wound characteristics, patient age, efficacy, cost, and patient preferences. In addition, special clothing in antibacterial and restraining fabric (leggings, tubular, socks, gloves, bodysuit) and without traumatizing seams are recommended.

A psychologist is involved to work alongside the dermatologist and nurse to support families from the earliest days of the newborn's life.

The pediatrician together with the dietician explain the importance of hydration, quality and quantity of nutritional intake, check the blood exams and prescribes support therapies with vitamins and food supplements. The endocrinologist often supports the role of the pediatrician in the management of complications (osteoporosis or vitamin deficiencies).

The physiotherapist teaches the advantages of physiotherapy and suggests the appropriate sport activities to prevent/delay contractures and osteoporosis. The dentist educates the parents to the oral hygiene since the first months of life in order to prevent/delay scarring, caries and parodontopathy. The plastic surgeon is involved in case of pseudosyndactyly or of squamous cell carcinoma. In patients with suspicion of oesophageal stricture/stenosis, the digestive surgeon is involved for specific diagnosis and treatment.

We offer to all families, as well as to the children and adolescents psychological support. Follow up of the patient depends on EB subtype and patient’s age to prevent and early treat complications. Thus, all EB patients and families get a direct contact to the referent nurse or dermatologist, avoiding normal waiting list.

### *Patients* < *5-years-old*

In Italy, a newborn affected with EB is immediately transferred to the specific referral center, often in Neonatal Intensive Care Unit, with a consequent dramatic effect on the parents due to the early child separation.

After birth, parents initially receive a diagnosis that is not definitive because EB includes multiple subtypes, among which some are benign, others are chronic and disabling, and others are fatal.

In addition, when parents see their child for the first time, skin lesions have a significant impact, which may cause an attitude of refusal and difficulty in breastfeeding the new-born. Moreover, in presence of blisters on the oral mucosa the new-born may have difficulties in feeding, and the support of specialized nursing staff, in order to support breastfeeding, has a crucial role from the first days of life (https://ern-skin.eu/tutorials/Epidermolysis bullosa and breastfeeding).

Therapeutic education before the child is discharged from the hospital is a key step in empowering both parents to manage their child at home. Starting from the first days, the medical staff helps the family in taking care of the new-born, from the most trivial daily activities, to advanced medications in order to make them autonomous.

It is fundamental to establish a relationship of continuity of care with the structures on the territory (primary care paediatrician). In our center, we carry out "protected discharges" ensuring, at least in severe cases, that through multidisciplinary meetings between the hospital staff and the primary care paediatrician the family is properly followed during the first weeks of life of the child. Indeed, the discharge represents a very difficult and drastic moment for the parents, that changes their perception of parenthood. The family must reconstruct its identity accepting the disease, reorganizing daily life, creating a routine of care in which parents should feel capable and effective, equipped to sustain the care by themselves. The parents of EB patients often face a multitude of emotional hardships while taking charge as caregivers. The feeling of joy of having a newborn turns into fear, guilt, shock, denial and helplessness because of the heavy burden of caring for a seriously ill child[9]. The family undergoes an important change after EB diagnosis, starting with the couple's relationship, which can later progress to other family members such as brothers or sisters. An early therapeutic alliance between the medical professionals and the family facilitates adaptation to the disease, which can stabilize and protect the family unit, the relationship between all members, and the psychophysical development of the child.

### *Patients* > *5–12 years-old*

As the child grows, parents must make continuous adjustments between the natural protective tendency (amplified by the disease), and the need to allow the child to explore the environment and to facilitate a progressive autonomy. Gradually the child begins to compare himself with others, and starts to ask questions often triggered by the reaction and curiosity of peers, which leads to a manifestation of a bad self-image.

The child becomes more aware of his or her illness, and experiences medication as a very traumatic moment. In the light of this, during the primary school phase (from 6 to 12 years old) we support the child to understand the disease and the fact that the potential complications could be reduced with good management. The child assumes more responsibility towards the pathology and above all begins to manage the disease, to have a well constructed self-perception, to understand the importance of medication and the application of topical therapy. Furthermore, he/she will be able to recognize and understand that taking care of himself/herself will make him/her feel better.

In this phase, the medical staff and the parents have a psychologically important role, helping the child the awareness of his/her condition, accepting and interpreting behavioural signals such as opposition to daily care, onset of signs of discomfort and answering to his/her direct questions. It is useful to involve the child so that he/she becomes part of the self-care, collaborating with his/her parents and becoming an "expert" in the disease [10].

### *Patients* > *12–18 years-old*

During this phase, the adolescents change their relationship with themselves and they have a full understanding of their condition, the characteristics of the disease, its rarity and chronicity. Bodily development, largely conditioned by the disease, does not correspond to the typical adolescent and does not allow for the investment process typical of this age. The omnipotence and natural expansion of the adolescent clashes with a body that is strongly limited and limiting.

At this stage, the wish for independence is frustrated by the need to remain connected to the family because of the disease and its associated demands.

The relationship with the healthcare staff, who increasingly speak directly to the child, can support him/her in the search for new motivations, reshaping the goals and negotiating with him/her the treatment, as far as possible.

In the adolescent period, it is very difficult to maintain adherence to the treatment, because they manifest a total rejection of their body, they realize that the pathology is chronic and worsening and they tend to be incompliant.

Furthermore, first complications (skin and mucosal cancer) begin to appear at this age, and treatment adherence is the only way to counteract or postpone these complications as much as possible.

## Results

Since our hospital is a pediatric center, we have included patients under 18 years of age; however, in the Table 1, we have reported 8 adult patients died due to squamous cell carcinoma (SCC) who contacted us for this specific complication.

**Table 1:** EB patients followed in Bambino Gesù Children’s Hospital 1990–2020

N		On follow-up	Died/cause	Lost to follow up	Occasionally transited
All patients	215	140 (65%)	27 (13%)	11 (5%)	37 (17%)
DEB^a^	138 (64%)	97 (45%)	9 (5%) *(RDEB*^b^*/SCC*^*c*^*)*	9 (4%)	23 (11%)
JEB	30 (14%)	9 (4%)	18 (8%) (*JEB-sev*^e^*/sepsis*)	2 (1%)	1 (0.5%)
EBS^f^	45 (21%)	32 (15%)	0	0	13 (6%)
KEB^g^	2 (1%)	2 (1%)	0	0	0

^a^*DEB,* Dystrophic epidermolysis bullosa*;* ^b^*RDEB,* Recessive DEB*;* ^c^*SCC,* Squamous cell carcinoma, ^d^*JEB,* junctional EB, ^e^*eJEB-sev,* JEB severe, ^f^*EBS,* EB simplex*,* ^g^*KEB,* Kindler EB

To date 215 patients with different EB subtypes have been followed in our hospital since 1990, among them 103 patients (48%) since birth. 140 patients (65%) are still followed by our team, 37 (17%) are followed at home; 27 patients (13%) died and 11 (5%) were lost to follow up (Table 1).

## Discussion

One positive outcome related to the adopted plan is that our patients manifested complications related to the disease subtype as expected and reported by the literature. Eighteen (8%) patients died during the first months of life due to recurrent sepsis and/or respiratory failure and were affected with JEB severe, while 9 (5%) affected with recessive dystrophic epidermolysis bullosa (RDEB) died for SCC in adulthood; these complications are well-known in each specific EB subtype especially for patients with RDEB and JEB, and percentages are in accordance with the reported data in the literature [11–14]. In EB patients, SCC usually arises in areas of chronic wounds and scars and the clinical presentation is frequently atypical with warty or ulcerated appearance. An early diagnosis is mandatory.

Clinical practice guidelines for the management of cutaneous SCC and epidemiological data on adult EB patients with SCC have been published [15–17]. However, only few case reports on paediatric EB patients with SCC have been reported [18, 19]. In our population one girl with RDEB, chronic wounds appeared at 11 years of age and she died at 16. This patient, for family reason, was lost to follow-up for 2 years after the diagnosis of the chronic wounds. No other pediatric patients developed squamous cell carcinoma.

Seven (3%) patients not born in Italy and came to our hospital for a specific requirement (e.g. diagnosis, genetic testing, wound care, disease management); 13 (6%) Italian patients affected with EBS localized have been followed initially and trained to manage their disease did not manifest complications requiring further consultation. Twelve Italian patients (5%) followed in another reference center consulted our team occasionally for a specific disease complication (e.g. oesophagus dilation, SCC surgery, pseudosyndactyly).

All foreigner patients were accompanied, upon our request, by a local caregiver (healthcare professional or parent) to be trained in our hospital for disease management and to take care about the patient in their own country. In some cases, the follow-up was maintained by e-mail.

## Conclusion

Therapeutic patient education is a continuous process of patient-centered medical care, enabling patients affected by chronic diseases, and their families, to better manage their illness: definition by the World Health Organization (WHO).

The process is dynamic considering the modification of disease clinical features, complications and consequent psychological impact during life. Making the patient and family feel welcomed, listened to, supported, and attended to with empathy becomes a facilitating factor for the well-being of all family members and their relationships. Certainly, EB subtypes and disease severity affect negatively the psychological involvement of the patient and his family. In addition, several factors may influence this aspect, such as patient age (adolescent), social environment, availability of a reference center, economical conditions. In our population, we did not assess the psychological impact, due to the lacking of an Italian validated questionnaire. Indeed recently, we have, in collaboration with DEBRA Italy, developed a specific questionnaire that we can use in the future as soon as it will be validated and published.

**Fig. 2:**
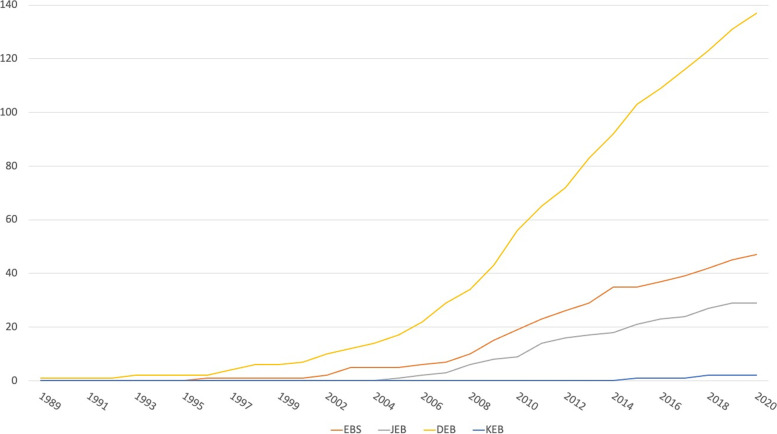
The graph shows the number of diagnoses according to the type of EB by year

The above-illustrated therapeutic plan adopted in all cases since the first approach, showed good clinical and psychological outcome in the major parts of our population. Indeed, the number of patients is continuously increasing: between 1990 and 2000, 8 patients with EB were admitted to our hospital, 75 patients between 2001 and 2010 and 132 patients between 2011 and 2020 (Fig. 2). Ninety-seven (45%) patients from other Italian regions are still on follow-up.

Altogether, an adequate disease multidisciplinary management, the involvement of both parents since the beginning of taking care, the constant psychological support and the appropriate and dynamic therapeutic patient education allow a greater adherence to treatment. Compliant child and parents let reduce the frequency of complications at an early stage and allow better quality of life and life expectancy. In keeping with our experience, a study was recently published describing the clinical, humanistic, and economic burden in EB patients, reporting that: (i) RDEB patients have experienced significant impact compared with other EB subtypes, (ii) disease management and decisions took into account the QoL of the patients, (iii) social activities were severely restricted, and finally (iv) humanistic and economic burden of RDEB extended beyond the patient affecting the family members .

During these 30 years of experience in our center, only 5% of patients with EB have been lost to follow-up and 5% died for SCC during the young adulthood (of which only one in adolescence, and the adults did not undergo a regular follow-up); this indicates that almost all patients joined to our therapeutic education plan adapted to age groups and patient-centred confirming that both family and patient feel accompanied along life.

The aim of our manuscript is to promote a multidisciplinary management including a dynamic therapeutic patient education throughout the life in order to reduce/delay the occurrence of complications, to avoid losing the patient at follow-up and to improve the quality of life of patients and their families.

The limits of this manuscript is that it is a retrospective study and it lacks from collected questionnaires from the patients and their parents on QoL. In addition, we did not consider other aspects where support to the family is also mandatory, such as school integration and disease costs.

## Data Availability

Family history, clinical and follow-up information were extracted from hospital electronic records. The datasets used and/or analysed during the current study are available from the corresponding author on reasonable request.

## Abbrevations

EB: epidermolysis bullosa
EBS: simplex epidermolysis bullosa
JEB: junctional epidermolysis bullosa
DEB: dystrophic epidermolysis bullosa
KEB: Kindler epidermolysis bullosa
IF: immunofluorescence
REBD: recessive dystrophic epidermolysis bullosa
SCC: squamous cell carcinoma
QoL: quality of life
